# Tau Reduction Does Not Prevent Motor Deficits in Two Mouse Models of Parkinson's Disease

**DOI:** 10.1371/journal.pone.0029257

**Published:** 2011-12-19

**Authors:** Meaghan Morris, Akihiko Koyama, Eliezer Masliah, Lennart Mucke

**Affiliations:** 1 Gladstone Institute of Neurological Disease, San Francisco, California, United States of America; 2 Biochemistry, Cellular and Molecular Biology Graduate Program, Department of Biological Chemistry, The Johns Hopkins University School of Medicine, Baltimore, Maryland, United States of America; 3 Departments of Neuroscience and Pathology, University of California San Diego, La Jolla, California, United States of America; 4 Department of Neurology, University of California San Francisco, San Francisco, California, United States of America; Brigham and Women's Hospital, Harvard Medical School, United States of America

## Abstract

Many neurodegenerative diseases are increasing in prevalence and cannot be prevented or cured. If they shared common pathogenic mechanisms, treatments targeting such mechanisms might be of benefit in multiple conditions. The tau protein has been implicated in the pathogenesis of diverse neurodegenerative disorders, including Alzheimer's disease (AD) and Parkinson's disease (PD). Tau reduction prevents cognitive deficits, behavioral abnormalities and other pathological changes in multiple AD mouse models. Here we examined whether tau reduction also prevents motor deficits and pathological alterations in two mouse models of PD, generated by unilateral striatal injection of 6-hydroxydopamine (6-OHDA) or transgene-mediated neuronal expression of human wildtype α-synuclein. Both models were evaluated on *Tau* ^+/+^, *Tau* ^+/–^ and *Tau* ^–/–^ backgrounds in a variety of motor tests. Tau reduction did not prevent motor deficits caused by 6-OHDA and slightly worsened one of them. Tau reduction also did not prevent 6-OHDA-induced loss of dopaminergic terminals in the striatum. Similarly, tau reduction did not prevent motor deficits in α-synuclein transgenic mice. Our results suggest that tau has distinct roles in the pathogeneses of AD and PD and that tau reduction may not be of benefit in the latter condition.

## Introduction

Neurodegenerative disorders are on the rise, most likely because greater longevity is increasing the age of populations around the world and age is a major risk factor for these conditions [1]–[3]. Alzheimer's disease (AD) and Parkinson's disease (PD) are the most prevalent of these disorders and no treatments are available to prevent, halt or reverse them. If these diseases had pathogenic mechanisms in common, drugs might be developed to target these mechanisms for the benefit of both patient groups. It is interesting in this regard that AD and PD overlap clinically. A proportion of patients show manifestations of both diseases [4]–[8], and first-degree relatives of patients with early-onset AD are at increased risk of developing PD [9]. The two diseases also overlap pathologically. A substantial proportion of AD patients have Lewy bodies in their brain, which are hallmarks of PD [10]–[12]. Conversely, a proportion of PD patients have amyloid plaques in their brain, which are hallmarks of AD [13], [14]. Both AD and non-demented PD patients have hyperphosphorylated tau [15]–[18], which aggregates in AD and in some cases of PD without dementia [19]–[25]. In addition, specific variants of the human tau (*MAPT)* gene appear to be genetic risk factors for PD [26]–[29].

Reduction of endogenous murine tau prevents cognitive deficits and various pathological alterations in several transgenic mouse models of AD [30]–[33]. Furthermore, neuronal overexpression of human α-synuclein in transgenic mice causes phosphorylation and aggregation of endogenous tau [18], [34]–[36]. In light of these findings and a recent report that tau reduction prevented dendritic degeneration in a neuronal culture model of mutant LRRK2-linked PD [37], we wondered whether tau reduction is also beneficial in mouse models of PD.

Human PD is characterized, among other things, by motor abnormalities such as slowed movements, rigidity, unstable posture and abnormal gait [38]. Pathologically, PD is characterized by loss of dopaminergic neurons in the substantia nigra, degeneration of their tyrosine hydroxylase (TH)-containing projections into the striatum and aggregation of α-synuclein into Lewy bodies [38].

Several of these abnormalities can be simulated in rodent models through administration of neurotoxins that target dopaminergic neurons or through neuronal expression of transgenes that encode relevant pathogenic proteins. Unilateral injection of 6-hydroxydopamine (6-OHDA) into the striatum of wildtype mice causes loss of TH-positive terminals in the striatum on the ipsilateral side and motor deficits [39]. This model can be used to address the question whether tau reduction protects dopaminergic neurons in the substantia nigra against neurotoxins and whether tau reduction can prevent motor deficits caused by dopaminergic cell loss. Either of these effects could be beneficial in PD. In a different PD-related model, neuronal expression of human wildtype α-synuclein (hSYN) causes motor deficits in transgenic mice [40]–[42]. This model can be used to address the question whether tau is necessary for α-synuclein-induced pathogenesis. Here, we evaluated both of these models on *Tau* ^+/+^, *Tau* ^+/–^ and *Tau* ^–/–^ backgrounds to determine whether tau reduction diminishes the severity of their motor deficits and pathological alterations.

## Materials and Methods

### Mouse Models

Sex-matched groups of 2.6–5.8-month-old Tau^+/+^, *Tau* ^+/–^, and *Tau* ^–/–^ mice [43] on a C57BL/6 background were used for striatal injections of 6-OHDA or vehicle. Heterozygous transgenic mice expressing hSYN directed by the Thy1 promoter on a mixed C57BL/6 and DBA/2 background [44] were crossed with *Tau* ^–/–^ C57BL/6 mice and their F1 offspring were intercrossed to generate mice with or without hSYN on the Tau^+/+^, *Tau* ^+/–^, or *Tau* ^–/–^ backgrounds. Only males were used for behavioral testing at 3.0–4.5 months of age. At the end of experiments, mice were deeply anesthetized with Avertin and killed by transcardial perfusion with saline. One or both hemibrains were fixed in 4% paraformaldehyde (from 32% solution, Electron Microscopy Services, PA) in phosphate buffer (pH 7.4). For hSYN mice, one hemibrain was frozen on dry ice. All experiments were approved by the Committee on Animal Research of the University of California, San Francisco (Approval Number AN085899-01A).

### 6-OHDA Injection

Mice were anesthetized, placed in a stereotaxic device and injected with either 2 µl containing 4 µg of 6-OHDA (Sigma, MO) in a solution of 2% L-ascorbic acid (Sigma, MO) in saline or the ascorbic acid-saline solution alone (vehicle). A single injection was performed in the striatum at the following coordinates relative to bregma: anterior-posterior  = +0.4, medial-lateral  = +2.0, dorsal-ventral  = −3.3. Injections were made at a rate of 0.5 µl/min with a 10-µl syringe (Hamilton Company USA, NV). Behavioral testing for acute effects of 6-OHDA began on the third day after injection and was completed within seven days. Behavioral testing to assess recovery began 24 days after the injection and was also completed within seven days.

### Open Field

A Flex-Field/Open Field Photobeam Activity System (San Diego Instruments, CA) was used to assess spontaneous movement. Mice were acclimated to the testing room for one hour before testing. Each mouse was placed in the center of a clear plastic chamber (41×41×30 cm) with two 16×16 photobeam arrays and allowed to explore for 15 min. Movements were recorded through the number of beam breaks and rearing was determined by beam breaks on the higher of the two arrays. The chamber was cleaned with 70% ethanol between mice. In our hands, hSYN mice at this age had no difference in total movements or rearing in the open field compared to nontransgenic mice, thus the tau-modulated hSYN cohort was not tested in the open field.

### Rota Rod

Mice were acclimated to the testing room for one hour before each session. Five mice were placed on the Rota Rod (Med Associates Inc, VT) for simultaneous testing and the computer recorded photobeam interruption when mice fell off the rotating rod. Photobeams were interrupted by the tester if the mouse held onto the rod without walking for three full rotations. Each mouse was given three trials with a maximum time on the Rota Rod of 300 seconds and with a 10-min rest between trials. The mice were first trained on the Rota Rod at a constant speed of 16 rpm. The next day they were trained on the Rota Rod in the morning at an accelerating speed of 4 rpm to 40 rpm over 300 s. In the afternoon, mice were tested on the Rota Rod at the same acceleration of 4 rpm to 40 rpm over 300 s and the average latency to fall off the Rota Rod was analyzed.

### Pole Test

The pole test was modified from previously reported protocols [41]. The pole consisted of a thin wooden dowel and a cross-shaped wooden base placed in a clean cage. Rubber bands were wrapped around the dowel at intervals of approximately 1.5 inches to increase traction. Mice were acclimated to the testing room for one hour before each session. Mice were placed at the top of the pole facing downwards and latency to descend the pole was measured. Trials were excluded if the mouse jumped or slid down the pole rather than climbed down. On the first day, each mouse was trained with two trials. On the second day, each mouse was given five trials and the lowest latency to descend the pole was analyzed.

### Gait Analysis

Gait analysis was done using the DigiGait software and treadmill (Mouse Specifics, Inc., MA). Mice were acclimated to the testing room for one hour before testing. Mice were placed on the treadmill and the belt speed was set at a constant pace of 15 cm/s. Mice were allowed to walk for several seconds until a regular gait pattern was observed; 4–5 seconds of gait video were then recorded. Mice were excluded if a regular gait pattern could not be recorded. Variable thresholds were set on the DigiGait software to allow the computer to identify and analyze the mouse paws from the recorded video clips. After the software analyzed each video, the data was manually curated to ensure that the analysis corresponded to actual paw placement. Any data that consistently misidentified paw placement on the belt was excluded from analysis.

### Hind Limb Clasp

The hind limb clasp test was performed as described [45]. Mice were acclimated to the testing room for one hour before testing. Each mouse was lifted by the tail and slowly lowered toward a surface for 10 s. The hind limbs were observed and each mouse was given a score for each trial. A score of 0 indicated that the hind limbs were extended for >50% of the trial period, a score of 1 indicated that one hind limb was retracted for >50% of the trial period, a score of 2 indicated that both hind limbs were partially retracted for >50% of the trial period, and a score of 3 indicated that both hind limbs were fully retracted and touching the abdomen for >50% of the trial period. Each mouse was tested three times and the scores were analyzed as described in the statistics section.

### Balance Beam

The balance beam consisted of two platforms connected by a removable plastic beam leading to an opaque box on top of one platform. Mice were acclimated to the testing room for one hour before testing. On the first day, mice were trained on a thick, round beam by placing them first a few inches from the box and leading them into the box, and then by placing them halfway across the beam and leading them into the box, if leading was required. The mice were then trained three times across the whole length of the beam with approximately 10 min between each trial. On the second day, mice were again trained three times on the thick beam. On the third day, the thick beam was replaced with a thin, square beam and the mice were tested three times. The average latency to cross the thin beam and the average number of times a foot slipped while mice crossed the beam during the testing sessions was analyzed. A trial was excluded from analysis if the mouse dragged its hind limbs across the beam for >50% of the distance. Acutely after 6-OHDA injection mice could not complete the balance beam test.

### Immunohistochemistry

Fixed hemibrains were sectioned by microtome (Leica Microsystems Inc., IL) into 30-µm sections, which were then stored at −20°C in a solution containing 30% glycerol (Fisher, PA), 30% ethylene glycol (Fisher, PA) and 40% phosphate-buffered saline (from 10× stock solution, Mediatech, Inc., VA) until immunostaining. Rabbit anti-TH (1∶2000 ab152, Abcam, MA) was used as the primary antibody. Primary antibody binding was detected with biotinylated donkey anti-rabbit (1∶500, Jackson Immunoresearch, PA), followed by avidin-biotin complex (Vector, CA). To determine the extent of the loss of striatal dopaminergic projections after 6-OHDA injection, the percent area occupied by TH immunoreactivity was determined with the percent area function of BIOQUANT (BIOQUANT Image Analysis Corporation, TN), in which the entire striatum was outlined by a variable region of interest and the percent area showing a staining signal above a set threshold was measured. Percent area on the injected side of the striatum was normalized to the percent area on the contralateral uninjected side. In hSYN transgenic mice, striatal TH immunoreactivity was quantitated by densitometry.

### Statistical Analysis

Investigators were blinded with respect to the genotype and treatment of mice during testing. Most measures were analyzed by two-way ANOVA and a Bonferroni post-hoc test with selected comparisons (Prizm, GraphPad Software, CA). Data points greater than two standard deviations from the mean for their group were excluded from analysis. In data where the variance between groups was severely unequal (Bartlett's test *p<*0.0001), the data was transformed by either a square-root, cube-root or log transformation to normalize variance between the groups. Whichever transformation best normalized variance was then analyzed by two-way ANOVA and a Bonferroni post-hoc test. All transformed data displayed equal variance across groups except rearing after the four-week recovery (Bartlett's test *p = *0.03). Two data sets were analyzed differently. The TH quantification was analyzed by one-way t-tests compared to 100% with a Benjamini-Hochberg p-value correction and by one-way ANOVA with a Bonferroni post hoc test to compare treated groups to each other. To assess genotype effects and genotype interaction in the hind limb clasp test, the raw score data was analyzed by probit regression with random effects taken into account (Stata, StataCorp LP, TX). Comparisons between individual genotype groups were made by Welch's t-test of the average hind limb clasp scores for each mouse with a Benjamini-Hochberg p-value correction for multiple comparisons.

## Results

### Tau Ablation Does Not Prevent Abnormalities Caused by 6-OHDA and Worsens Some of Them

**Figure 1:**
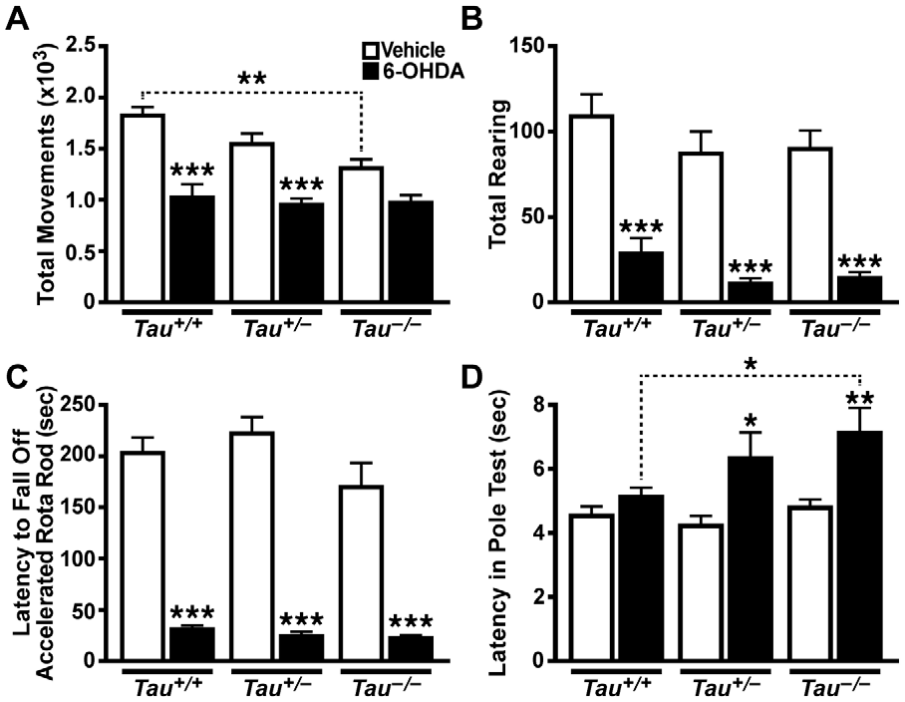
Tau Reduction Does Not Prevent Motor Deficits Induced by Acute Striatal 6-OHDA Injection. Mice (n = 9–12 per treatment and genotype) received a unilateral striatal injection of 6-OHDA or vehicle at 2.6–5.8 months of age and were tested behaviorally beginning 3 days later. A) Total movements in the open field were reduced by 6-OHDA treatment and by tau reduction (*p*<0.0001 for treatment effect, *p* = 0.01 for genotype effect, and *p* = 0.054 for interaction by two-way ANOVA). B) Rearing in the open field was reduced by 6-OHDA regardless of tau levels (*p*<0.0001 for treatment effect, *p* = 0.09 for genotype effect, and *p* = 0.15 for interaction by two-way ANOVA after cube-root transformation). C) Latency to fall off an accelerating Rota Rod was reduced by 6-OHDA but not by tau reduction (*p*<0.0001 for treatment effect, *p* = 0.40 for genotype effect, and *p* = 0.22 for interaction by two-way ANOVA after cube-root transformation). D) Latency to descend in the pole test was increased by 6-OHDA and by tau reduction (*p*<0.0001 for treatment effect, *p* = 0.05 for genotype effect, and *p* = 0.11 for interaction by two-way ANOVA). *p<0.05, **p<0.01, ***p<0.0001 vs. vehicle-treated mice of same *Tau* genotype or as indicated by bracket (Bonferroni test with selected comparisons of vehicle vs. 6-OHDA treatment within each genotype, and *Tau* ^+/+^ vs. *Tau* ^–/–^ for each treatment). Error bars represent SEM.

To evaluate the effects of tau reduction acutely and after recovery from 6-OHDA injection, *Tau^+/+^*, *Tau* ^+/–^ and *Tau* ^–/–^ mice received unilateral injections of 6-OHDA into the striatum and were examined for motor abnormalities in the open field, Rota Rod and pole tests beginning 3 days (Fig. 1) and 24 days (Fig. S1) thereafter. Acutely, 6-OHDA injected mice of all three genotypes showed reduced movements and rearing in the open field (Fig. 1A,B) and reduced latency to fall off the accelerating Rota Rod (Fig. 1C). 6-OHDA increased the latency to descend the pole in the pole test only in mice with reduced tau levels, but not in wildtype mice (Fig. 1D). In vehicle injected mice, tau ablation reduced activity in the open field (Fig. 1A) but did not significantly affect performance in the other tests (Fig. 1B–D).

After the recovery period, all groups of mice showed a similar level of activity in the open field (Fig. S1A). Tau ablation also had no effects on recovery of motor functions in the other tests at this stage, with 6-OHDA injected *Tau* ^+/+^ and *Tau* ^–/–^ mice showing no significant differences in rearing, Rota Rod fall latency or pole descent latency (Fig. S1B–D).

**Figure 2:**
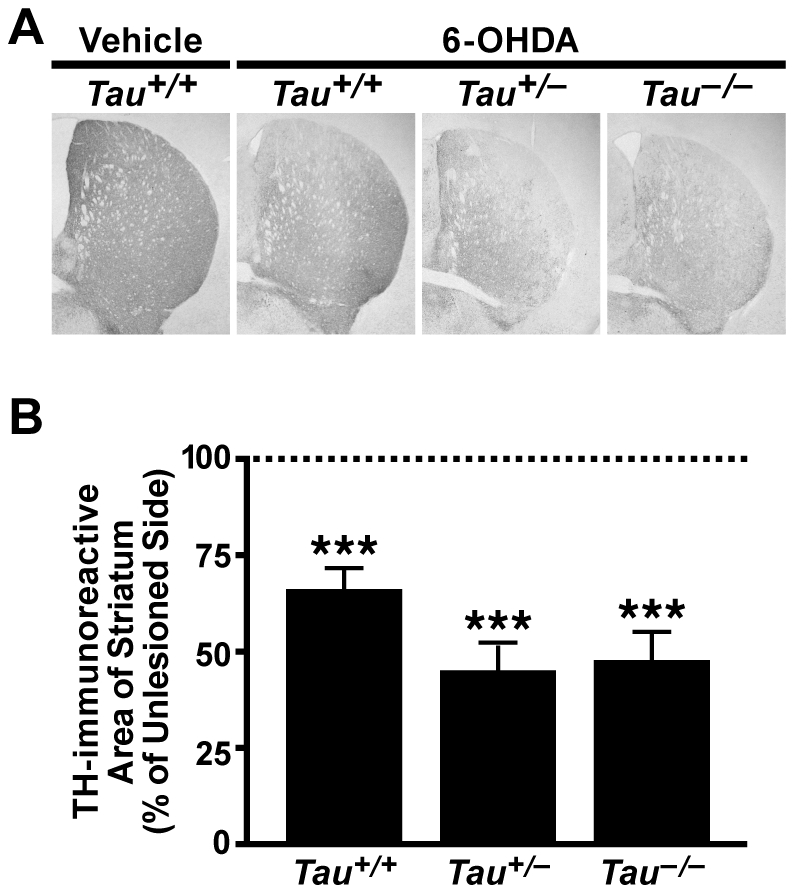
Tau Reduction Does Not Prevent Loss of TH after Striatal Injection of 6-OHDA. Mice (n = 7-12 per treatment and genotype) received a unilateral striatal injection of 6-OHDA or vehicle at 2.6–5.8 months of age and were analyzed by tyrosine hydroxylase immunohistochemistry and light microscopy 50 days later. A) Representative photomicrographs from a vehicle-injected wildtype mouse and 6-OHDA injected mice of different *Tau* genotypes demonstrating loss of TH immunoreactivity in the striatum. B) The effect of 6-OHDA was quantitated by expressing the area of TH immunostaining on the lesioned side as a percentage of that on the unlesioned side of the brain. 6-OHDA caused loss of TH immunoreactivity in the striatum and there was a trend toward greater losses in groups with reduced tau levels. ***p<0.0001 vs. 100% (no loss of dopaminergic projections) by one-sample t-test with a Benjamini-Hochberg correction. One-way ANOVA between groups showed no significant changes. Error bars represent SEM.

To assess the effect of 6-OHDA on striatal projections from dopaminergic neurons in the substantia nigra, we immunostained brain sections from the behaviorally tested mice for TH (Fig. 2A). Vehicle treated mice showed no loss of TH staining (Fig. 2A, quantification not shown). 6-OHDA treatment caused a loss of striatal TH in all three genotypes, and there was a strong trend towards increased TH losses in 6-OHDA injected mice with reduced tau levels (Fig. 2B). Thus, tau ablation does not prevent, and may partly enhance, acute motor and pathological deficits induced by 6-OHDA injection.

### Tau Reduction Does Not Prevent Motor Deficits in Human Wildtype α-Synuclein Transgenic Mice

**Figure 3:**
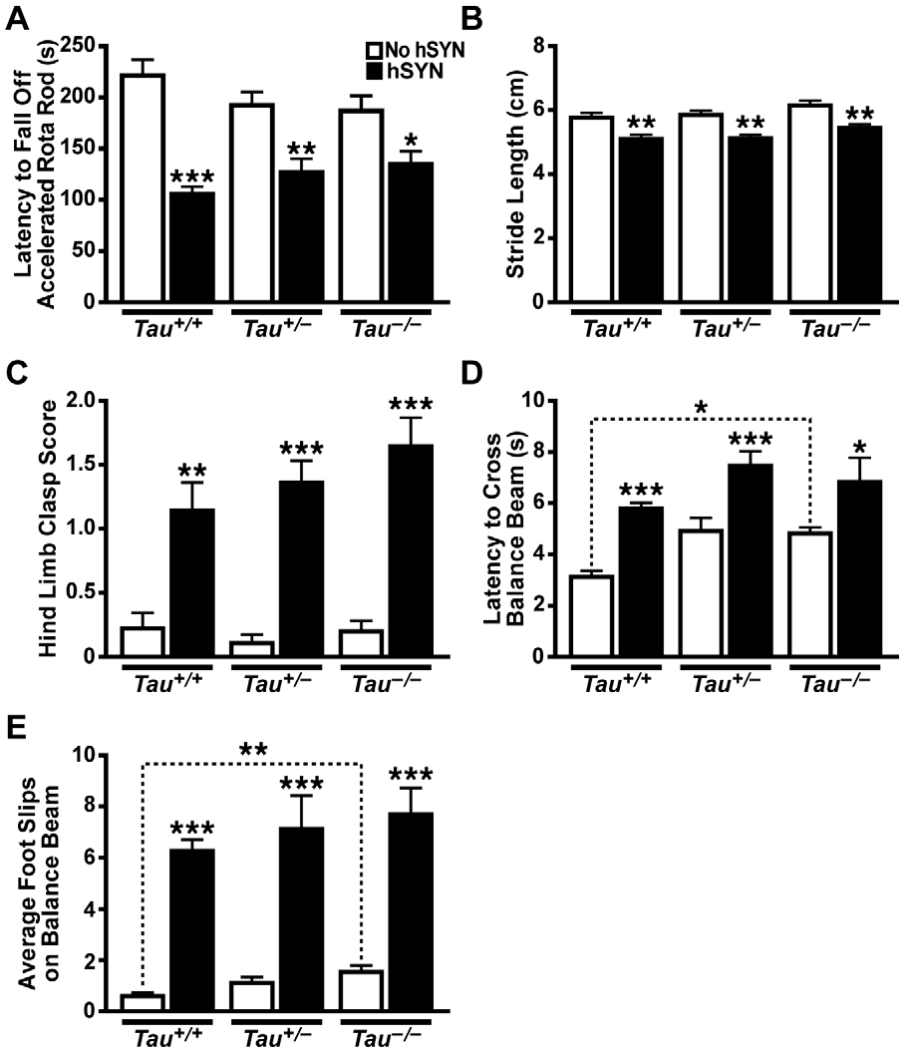
Tau Reduction Does Not Prevent Motor Deficits in hSYN Transgenic Mice. hSYN mice on different *Tau* backgrounds (n = 12–15 per group) were analyzed behaviorally at 3.0–4.5 months of age. A) Transgenic expression of hSYN impaired performance on an accelerating Rota Rod, reducing fall latencies. Tau reduction was associated with non-significant trends towards improved fall latencies in mice with hSYN and towards impaired fall latencies in mice without hSYN (*p*<0.0001 for hSYN effect, *p* = 0.96 for *Tau* effect, and *p* = 0.04 for genotype interaction by two-way ANOVA). B) hSYN mice had shortened stride lengths regardless of *Tau* genotype (*p*<0.0001 for hSYN effect, *p* = 0.014 for *Tau* effect, and *p* = 0.97 for interaction by two-way ANOVA). C) hSYN mice showed prominent increases in hind limb clasp reflex and tau reduction showed a non-significant trend to worsen this abnormality (*p*<0.001 for hSYN effect, *p* = 0.45 (*Tau* ^+/–^) and *p* = 0.91 (*Tau^–/–^)* for *Tau* effects, and *p* = 0.46 (*Tau* ^+/–^) and *p = *0.48 (*Tau* ^–/–^) for hSYN interaction by probit regression). D, E) Transgenic expression of hSYN and tau reduction both increased (D) the latency to cross a balance beam (*p*<0.0001 for hSYN effect, *p* = 0.07 for *Tau* effect, and *p* = 0.94 for interaction by two-way ANOVA) and (E) the number of foot slips while crossing a balance beam (*p*<0.0001 for hSYN effect, *p* = 0.022 for *Tau* effect, and *p* = 0.19 for interaction by two-way ANOVA after square root transformation). Between 33–54% of mice in each hSYN group had to be excluded from balance beam analysis because they dragged their hind limbs across the beam. *p<0.05, **p<0.01, ***p<0.0001 vs. mice without hSYN on the same *Tau* genotype or as indicated by bracket (Bonferroni test (A, B, D, E) or Welch's t-test with a Benjamini-Hochberg correction (C) for selected comparisons of + vs. – hSYN for each *Tau* genotype and *Tau^+/+^* vs. *Tau* ^–/–^ for each hSYN genotype). Error bars represent SEM.

Mice with neuronal expression of hSYN directed by the Thy1 promoter (hSYN mice) were crossed onto the *Tau* ^+/+^, *Tau* ^+/–^ or *Tau* ^–/–^ background. Motor functions of the resulting offspring were assessed at 3.0–4.5 months of age. Independent of *Tau* genotype, hSYN expression caused abnormalities in fall latency in the Rota Rod test (Fig. 3A), stride length (Fig. 3B), hind limb clasp reflex (Fig. 3C), latency to cross a balance beam (Fig. 3D) and foot slips on the balance beam (Fig. 3E). Tau reduction did not significantly modulate these effects (Fig. 3A–E). In mice without hSYN, tau ablation increased latency to cross and foot slips on the balance beam (Fig. 3D and E), but had no significant effect on the other measures.

To assess the effect of tau ablation on the pathology of SYN mice, we examined striatal TH staining in the behaviorally tested mice. Compared with wildtype mice, hSYN mice showed no decreases in striatal TH staining on any of the *Tau* backgrounds (data not shown), consistent with previous findings in hSYN/*Tau* ^+/+^ mice at this age [46].

## Discussion

These findings demonstrate that tau reduction does not protect mice against motor deficits and pathological alterations caused by striatal injection of 6-OHDA or transgene-mediated neuronal expression of hSYN. Thus, wildtype murine tau does not appear to contribute causally to PD-like motor deficits and pathological alterations in these models. In contrast, endogenous tau is required for amyloid-β (Aβ) peptides to impair neurons in primary cultures [47]–[49] and in human amyloid precursor protein (hAPP) transgenic mice [30], [32], [33]. It is also needed for apolipoprotein E4, the most important genetic risk factor for AD, to cause cognitive decline and neuronal loss in knockin mice [31]. However, tau reduction did not alter the age of disease onset or mortality in a mouse model of amyotrophic lateral sclerosis [33], providing further support for the conclusion that tau specifically contributes to functional and pathological abnormalities in experimental models of some neurodegenerative disorders but not of others.

It is important to consider the limitations of the mouse models used in this study and the extent to which one can extrapolate from these models to human PD. Because the 6-OHDA model is caused by an acute insult, it probably does not simulate the etiology of sporadic PD, a notoriously chronic condition. However, it is a robust model of dopaminergic cell loss and of motor deficits resulting from deficient dopaminergic input to the striatum, two cardinal features of PD. Our data suggest that wildtype tau does not enable the neural network dysfunction that underlies such motor deficits. This result was not predictable as tau enables neuronal hypersynchrony in models of AD [33] and neuronal hypersynchrony has also been implicated in the pathophysiology of PD [50], [51]. The differential effects of tau reduction in models of AD versus PD suggest that tau plays distinct roles in their pathogenic mechanisms. The hSYN mice used in this study model α-synuclein-induced behavioral alterations and may be relevant to the early pathogenesis of PD and other synucleinopathies. However, they do not replicate PD-like neurodegeneration in the substantia nigra at this age [52], possibly due to low α-synuclein expression levels in this structure. Our results indicate that tau does not enable or mediate early α-synuclein-induced motor deficits in this model.

Notably, we cannot exclude a pathogenic role of tau in humans with PD or other synucleinopathies or that tau reduction might be of benefit in specific forms of PD, for example, those caused by LRRK2 mutations [37]. It is therefore interesting to comment on potential risks of tau reduction beyond lack of therapeutic benefit in forms of PD simulated by the models examined in the current study. Of all the functional outcome measures we evaluated in 6-OHDA injected mice and untreated hSYN mice, tau reduction significantly worsened only one: descent latency of 6-OHDA injected mice in the pole test (Fig. 1D), suggesting that the risk of enhancing PD-like alterations by tau reduction may be low. Tau reduction also had rather subtle effects on pathological measures in the PD models analyzed here. Although tau reduction appeared to exacerbate the 6-OHDA-induced loss of TH immunoreactivity in the striatum, this trend did not reach statistical significance.

Furthermore, tau reduction *per se* had relatively subtle effects in only two of our behavioral tests in vehicle injected controls or untreated mice lacking hSYN. In vehicle injected mice, tau reduction was associated with decreased total movements in the open field acutely after the injection (Fig. 1A), but not after a three week recovery period (Supp. Fig. 1A). In untreated mice lacking hSYN, tau reduction impaired performance on the balance beam (Fig. 3D, E). However, these impairments were much less severe than those caused by hSYN expression (*p = *0.009 for balance beam latency and *p<*0.0001 for foot slips comparing *Tau^–/–^* with hSYN/*Tau^+/+^* mice by two-tailed t-test). Thus, tau reduction was well tolerated overall and caused only minimal motor deficits.

Because tau reduction effectively prevented AD-like abnormalities in hAPP transgenic mice but not PD-like deficits in the models analyzed here, it is tempting to speculate that tau plays different roles also in the human conditions and that tau reduction might be beneficial in AD, but not in the most common forms of PD. Additional studies are needed to further test these hypotheses and to further explore the potential therapeutic value of this strategy in different neurological conditions.

## Supporting Information

Figure S1 Tau Reduction Does Not Alter Recovery of Motor Function after 6-OHDA Injection. Mice (n = 7-12 per genotype and treatment) received a unilateral striatal injection of 6-OHDA or vehicle at 2.6–5.8 months of age and were tested behaviorally beginning 24 days later. A) Total movements in the open field were similar in all groups. B) Rearing in the open field was reduced by 6-OHDA and this abnormality was improved by tau ablation (*p* = 0.003 for treatment effect, *p* = 0.04 for genotype effect, and *p* = 0.44 for interaction by two-way ANOVA after log transformation). C) Fall latency on the accelerated Rota Rod was decreased by 6-OHDA treatment regardless of *Tau* genotype (*p*<0.0001 for treatment effect, *p* = 0.71 for genotype effect, and *p* = 0.15 for interaction). D) Latency to descend in the pole test was increased by 6-OHDA mostly in *Tau* ^+/–^ mice (*p*<0.0001 for treatment effect, *p* = 0.04 for genotype effect, and *p* = 0.09 for interaction). *p<0.05, **p<0.01, ***p<0.0001 vs. vehicle-treated mice of same *Tau* genotype or as indicated by bracket (Bonferroni test with selected comparisons as in Fig. 1). Error bars represent SEM.
(TIF)
