# Supplementary figures and images for: Tau Reduction Does Not Prevent Motor Deficits in Two Mouse Models of Parkinson's Disease
Source: PLoS One. 2011 Dec 19;6(12):e29257. doi: 10.1371/journal.pone.0029257 (PMC3242771; doi:10.1371/journal.pone.0029257)

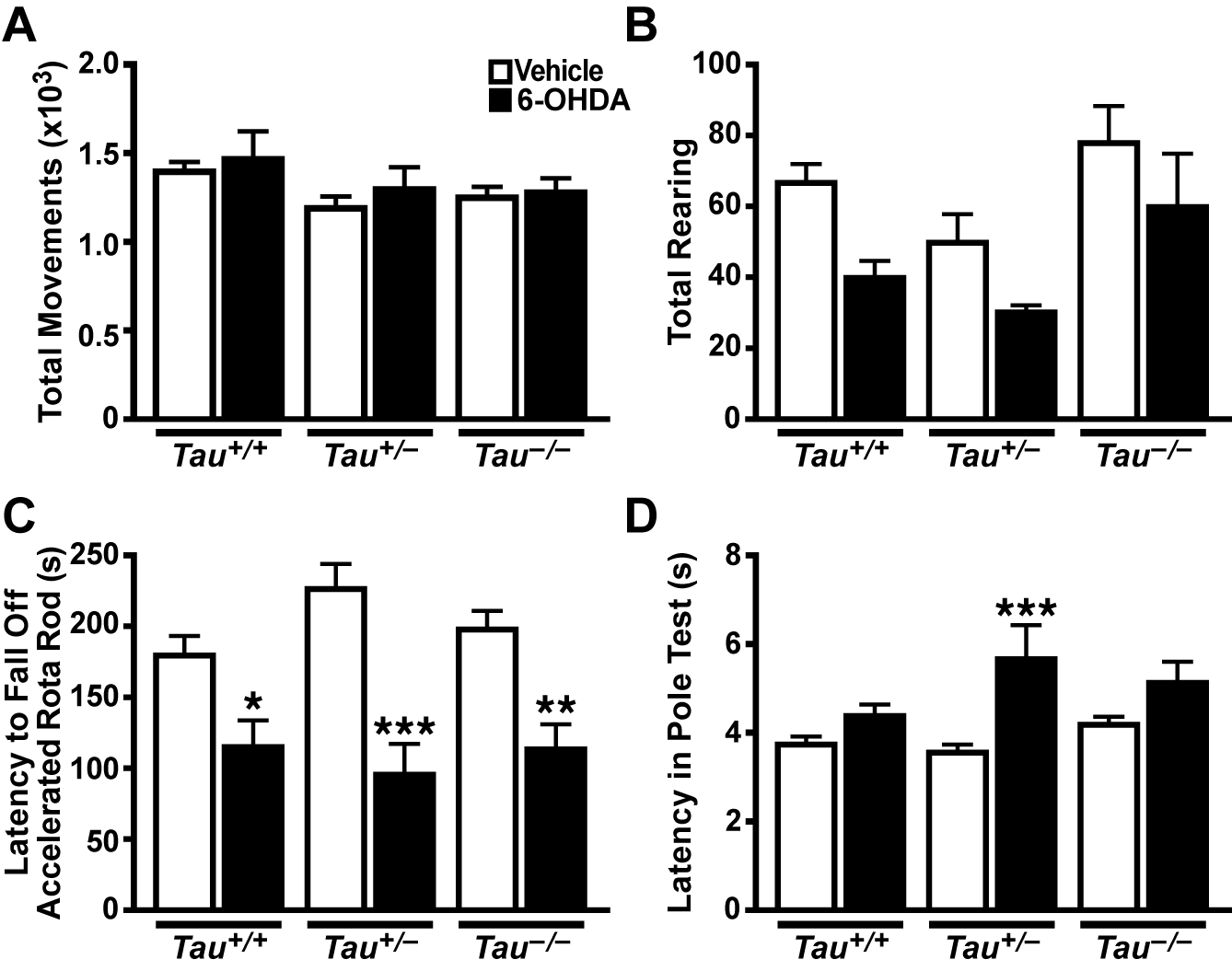

Supplement: Figure S1 — Tau Reduction Does Not Alter Recovery of Motor Function after 6-OHDA Injection. Mice (n = 7-12 per genotype and treatment) received a unilateral striatal injection of 6-OHDA or vehicle at 2.6–5.8 months of age and were tested behaviorally beginning 24 days later. A) Total movements in the open field were similar in all groups. B) Rearing in the open field was reduced by 6-OHDA and this abnormality was improved by tau ablation (p = 0.003 for treatment effect, p = 0.04 for genotype effect, and p = 0.44 for interaction by two-way ANOVA after log transformation). C) Fall latency on the accelerated Rota Rod was decreased by 6-OHDA treatment regardless of Tau genotype (p<0.0001 for treatment effect, p = 0.71 for genotype effect, and p = 0.15 for interaction). D) Latency to descend in the pole test was increased by 6-OHDA mostly in Tau +/– mice (p<0.0001 for treatment effect, p = 0.04 for genotype effect, and p = 0.09 for interaction). *p<0.05, **p<0.01, ***p<0.0001 vs. vehicle-treated mice of same Tau genotype or as indicated by bracket (Bonferroni test with selected comparisons as in Fig. 1). Error bars represent SEM. (TIF) [file pone.0029257.s001.tif]
